# Seroprevalence of spotted fever group (SFG) rickettsiae infection in domestic ruminants in Khartoum State, Sudan

**DOI:** 10.1002/vms3.59

**Published:** 2017-02-21

**Authors:** Nagwa M. Eisawi, Dina A. Hassan, Mohammed O. Hussien, Azza B. Musa, Abdel Rahim M. El Hussein

**Affiliations:** ^1^ Central Laboratory Ministry of Higher Education and Scientific Research P.O. Box 7099 Khartoum Sudan; ^2^ Central Veterinary Research Laboratory (CVRL) Animal Resources Research Corporation (ARRC) P.O. Box 8067 El Amarat, Khartoum Sudan

**Keywords:** SFG rickettsiae, domestic ruminants, Sudan, IFAT, risk factors

## Abstract

Spotted fever group (SFG) rickettsiosis is caused by obligatory intracellular Gram‐negative bacteria that belong to the genus *Rickettsia*. Ticks belonging to the family Ixodidae can act as vectors, reservoirs or amplifiers of SFG rickettsiae. This study was conducted to estimate the seroprevalence of SFG rickettsioses in cattle, sheep and goats from Khartoum State, Sudan. Blood samples were collected from a total of 600 animals (sheep, goats and cattle) from 32 different farms distributed in three locations in Khartoum State during the period January to December 2012. Sera were tested for antibodies against SFG rickettsiae using IFAT. The prevalence of seropositivity was 59.3% in sheep, 60.1% in goats and 64.4% in cattle. Season was significantly (*P < *0.05) associated with seroprevalence of SFG rickettsiae in cattle during winter. The SFG rickettsiae antibodies prevalence was significantly higher in female compared with male in sheep, but there were no significant differences between male and female in either cattle or goats. The prevalence was significantly higher in adult animals compared with young in both sheep and goats. With regard to management system, there was a significant difference in the prevalence in cattle raised in closed system compared with those raised in semi‐intensive system. In contrast, there was significant difference in the seroprevalence of SFG in sheep where the prevalence was higher in the sheep raised in semi‐intensive system compared with those raised in close system. There was no significant difference in the seroprevalence in goats with regard to management systems. The unexpected high prevalence of SFG rickettsia antibodies in domestic ruminants sera suggest that the veterinary and public health impact of these agents in Sudan need further evaluation especially in humans.

## Introduction

Spotted fever group (SFG) rickettsiosis is caused by obligatory intracellular Gram‐negative bacteria that belong to the genus *Rickettsia* (Parola *et al*. 2005). Ticks belonging to the family Ixodidae can act as vectors, reservoirs or amplifiers of SFG rickettsiae. These bacteria do not normally infect humans during their natural cycles between their arthropod and vertebrate hosts. Ecological characteristics of the tick vectors influence the epidemiology and clinical aspects of tick‐borne diseases (Parola & Raoult 2001). Some rickettsiae, such as *Rickettsia rickettsii*, may be associated with several tick vectors from different genera (Parola *et al*. 2005). This contrasts with other rickettsiae, such as *Rickettsia conorii*, which appear to be associated with only one tick vector (Raoult & Roux 1997). Between these extremes, there are certain rickettsiae which are associated with several species within the same genus, such as association of *Rickettsia africae* and *Rickettsia slovaca* with *Amblyomma* spp. and *Dermacentor* spp., respectively (Parola & Raoult 2001). In sub‐Saharan Africa, the main causative agents of SFG rickettsiae include *R. conorii*,* R. africae*,* R. aeschlimannii*,* R*. *sibirica* and *R. massiliae* (Parola 2006). The indirect immunofluorescence assay (IFA) is currently the test of choice for the serological diagnosis of rickettsial infection in humans and animals (Yu & DH 2003; Horta *et al*. 2004). However, the cross reactivity of IFA does not allow for identification of the infecting *Rickettsia* species. The geographic origin of the infection has been one of the best indicators of species identity (Horta *et al*. 2004). Testing a clinical serum sample against the possible *Rickettsia* species known to occur in a given area is ideal, because often homologous antibody titres are higher than heterologous antibody titres. In some cases, the differences in titres may be great enough to differentiate among the rickettsial species potentially stimulating the immune response (LaScola & Raoult 1997). A high proportion (80–100%) of cattle in endemic areas has serological evidence of exposure to SFG rickettsiae (Kelly *et al*. 1991). High seroprevalence (87%) has also been reported in goats (Parola *et al*. 1999), although in another study, seroprevalence rates in the three common domestic ruminants (cattle, goats and sheep) reflected the host preference of the vector *Amblyomma variegatum*, with the highest prevalence in cattle and much lower prevalence in goats and sheep (Kelly *et al*. 2010). A previous molecular detection of SFG rickettsia in *Hyalomma* spp. and *Amblyomma* spp. (*A. lepidum*,* A. variegatum*,* H. dromedarii*,* H. truncatum*,* H. marginatum*) collected in Sudan demonstrated that SFG rickettsia were highly prevalent in these ticks (Morita *et al*. 2004; Nakao *et al*. 2015). Entomological studies performed in Khartoum State confirmed that Ixodid ticks, the potential vectors of SFG rickettsia, are highly prevalent in domestic animals and that the close contact of humans with ticks is continual and permanent (Mohamed *et al*. 2004). Observations reported by Mohamed *et al*. (2004) indicated that seven species of Ixodidae ticks were infesting cattle in Khartoum State. *Hyalomma anatolicum* constituted 70.6% and 83.3% of the tick fauna in Soba and Kuku, while *Rhipicephalus e. evertsi* constituted 22.5% of the tick fauna in Soba and 6.2% in Kuku (Mohamed *et al*. 2004). Osman (1999) collected *H. anatolicum*,* R. e. evertsi* and ticks of the *R. sanguineus* group from sheep in the Khartoum University Farm. The aim of this study was to estimate the seroprevalence of SFG rickettsioses in cattle, sheep and goats from Khartoum State, Sudan.

## Materials and methods

### Study area

**Figure 1:**
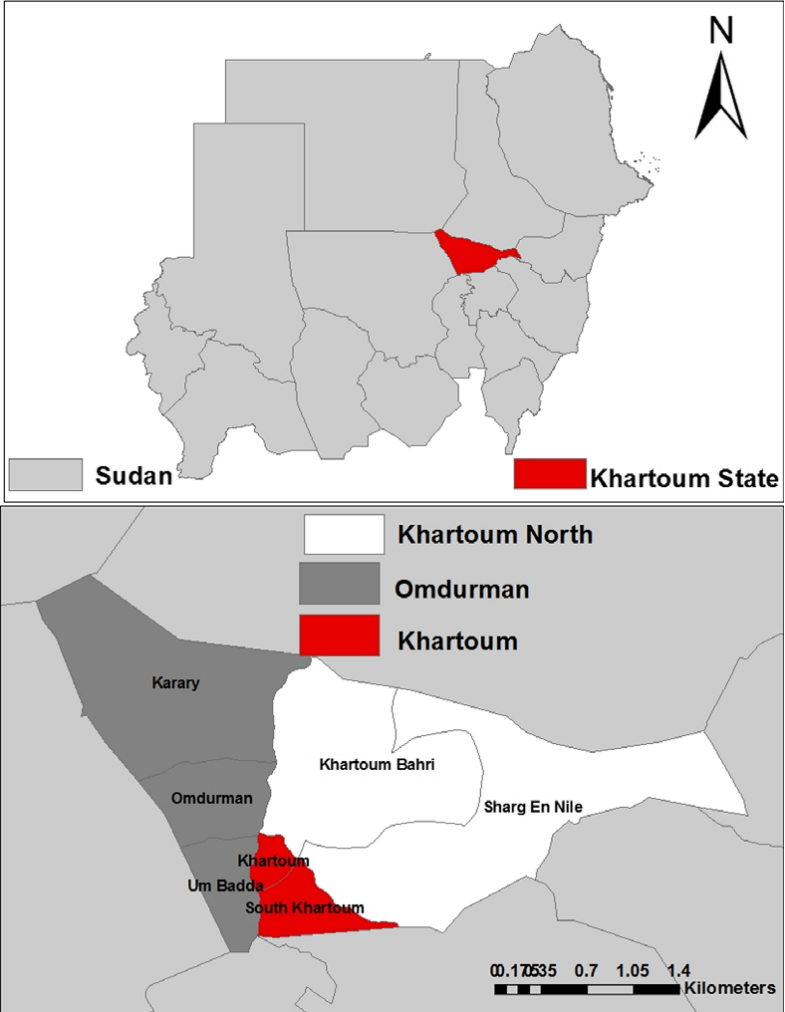
Map of Khartoum State showing locations where samples were collected.

The survey was conducted during the period from January to December 2012 in three locations (Khartoum, Omdurman and Khartoum North) in Khartoum State in the poor savannah climatic zone, Sudan (15.16–16.65° North and 31.64–32.05° East) (Fig. 1).

### Study design

This is a cross‐sectional survey that was carried out in three locations in Khartoum State. Sample size estimation of the animals was calculated using the following formula (Thrusfield 2007):

$$n=z^{2}PQ/L^{2}.$$

where *n* is the required number of individuals to be examined; *z* is a constant = 1.96; *P* is a known or estimated prevalence; *Q* = (1 − *P*) and *L* are the allowable error. As the prevalence of SFG rickettsiae in different regions of the Sudan has never been substantially determined, this study assumed an expected prevalence of SFG rickettsiae in domestic ruminants to be 50% (Thrusfield 2007). The number of animals estimated using this formula was 384. The sample size was increased to 600, in order to allow for more reliable statistical analysis.

### Blood collection

The investigation was carried out in compliance with the animal welfare code of Sudan. Six hundred blood samples from sheep, goats and cattle (200 from each species) from 32 farms distributed in the three locations in Khartoum State were collected. Five ml jugular venous blood was collected from each animal in a plain container. Sera were separated by centrifugation at 1500 rpm for 10 min and then stored at −20°C until tested.

### Immunofluorescent antibody test for detection of SFG rickettsial infection

**Figure 2:**
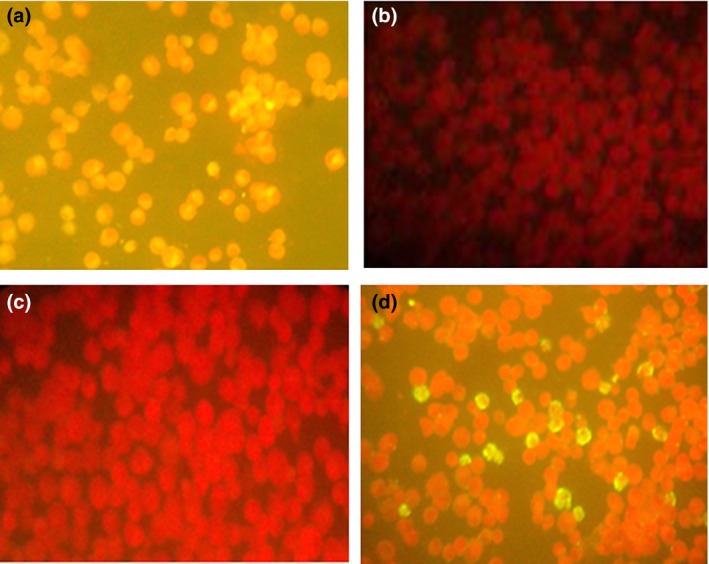
IFAT results for SFG rickettsiae: (a) positive control, (b) negative control, (c) negative sample and (d) positive sample.

Rickettsia SFG antibodies were detected by indirect fluorescent antibody test (IFAT) using different species IgG conjugates (Fuller Laboratories, Fullerton, California, USA). Twelve‐well masked slides containing acetone‐fixed Vero cells, some of which were infected with the bitterroot strain of *R. rickettsii* (chemically killed) were used. The procedure was performed according to the manufacturer's instructions (Raoult & Dasch 1989) (Fig. 2). Serum was considered to contain antibodies against the rickettsiae if it displayed a reaction at the 1:64 dilution.

### Statistical analysis

Data were compared using Pearson's chi‐squared test. A *P* ≤ 0.05 was considered indicative of a statistically significant difference. All the data analyses were performed using the SPSS 20.0 for Windows software package (SPSS, Chicago, IL, USA).

## Results

The prevalence of SFG antibodies was found to be 64.4%, 60.1% and 59.3% in cattle, goats and sheep, respectively (Table 1). There was no significant difference in prevalence of SFG rickettsiae antibodies among different animal groups. The seasonally related prevalence of SFG rickettsiae antibodies was significantly (*P *=* *0.027) higher in cattle during winter season. The SFG rickettsiae antibodies rate was significantly (*P *=* *0.02) higher in female compared with male in sheep, and in older animals compared with young ones in both sheep (*P *=* *0.04) and goats (*P *=* *0.000). According to location, the infection rate was significantly (*P *=* *0.01) higher in Khartoum North in goats. According to the management systems there was significant (*P *=* *0.02) difference in the prevalence in cattle raised in closed system compared to those raised in semi‐intensive management system. In contrast, there was significant (*P *=* *0.000) difference in the prevalence of SFG antibodies in sheep raised in semi‐intensive management system compared with those raised in close system (Tables 2, 3, 4).

**Table 1:** Seroprevalence of spotted fever group in domestic ruminants

Animal type	Positive	Negative	*P*
Cattle	125 (64.4%)	69 (35.6)	0.383^a^
Sheep	118 (59.3%)	81 (40.7%)	
Goats	119 (60.1%)	79 (39.9%)	

a No significant differences in seroprevalence rate were found among the three kinds of animals tested.

**Table 2:** Influence of certain risk factors on seroprevalence of spotted fever group in cattle

Risk factor	Group	No. positive	No. negative	Percentage positive	*P*
Season	Summer†	16	9	64.0	0.027*
	Winter‡	109	60	64.5	
Age (years)	<3	26	11	70.3	0.121
	3–6	38	27	58.5	
	6.1–9	41	27	60.3	
	>9	20	4	83.3	
Location	Khartoum	38	23	62.3	0.13
	Omdurman	44	15	74.6	
	Khartoum North	43	31	58.1	
Sex	Male	16	7	69.6	0.58
	Female	109	62	63.7	
Management	Intensive	105	48	68.6	0.02*
	Semi‐intensive	20	21	48.8	

*P < 0.05. ^†^March–June. ^‡^November–February.

**Table 3:** Influence of certain risk factors on seroprevalence of spotted fever group in sheep

Risk factor	Group	No. positive	No. negative	Percentage positive	*P*
Season	Summer†	36	19	65.5	0.36
	Winter‡	44	38	53.7	
	Autumn§	38	24	61.3	
Age (years)	<1	25	30	45.5	0.04*
	1–4	58	35	62.4	
	>4	35	16	68.6	
Location	Khartoum	37	26	58.7	0.2
	Omdurman	26	26	50.0	
	Khartoum North	55	29	65.5	
Sex	Male	46	45	50.5	0.02*
	Female	72	36	66.7	
Management	Intensive	86	76	53.1	0.000*
	Semi‐intensive	32	5	86.5	

*P < 0.05. ^†^March–June. ^‡^November–February. ^§^July–September.

**Table 4:** Influence of certain risk factors on seroprevalence of spotted fever group in goats

Risk factor	Group	No. positive	No. negative	Percentage positive	*P*
Season	Summer†	41	16	71.9	0.09
	Winter‡	49	37	57.0	
	Autumn§	29	26	52.7	
Age (years)	<1	24	35	40.7	0.000*
	1–3	38	30	55.9	
	>3	57	14	80.3	
Location	Khartoum	27	30	47.4	0.01*
	Omdurman	31	26	54.4	
	Khartoum North	61	23	72.6	
Sex	Male	24	14	63.2	0.67
	Female	95	65	59.4	
Management	Intensive	115	79	59.3	0.1
	Semi‐intensive	4	0	100	

*P < 0.05. ^†^March–June. ^‡^November–February. ^§^July–September.

## Discussion

In the present study, the seroprevalence of SFG rickettsiae using IFAT was found to be 59.3%, 60.1% and 64.4% in sheep, goats and cattle, respectively, comparable with that reported in Guadeloupe in the French, West Indies, in which the seroprevalence of *R. africae* was 55.3% in cattle at a titre ≥ 1:100 and 63.3% in goats at a titre ≥ 1:100 (Parola *et al*. 1999). However, our results were in contrast with 15.7% and 65% found in sheep and goats, respectively, in Catalonia (Ortuno *et al*. 2012). Also, the seroprevalence of SFG rickettsiae reported in domestic ruminants in this study is higher than that reported for goats (20%) and cattle (30%) in Sri Lanka and for sheep (7.5%) in Tanzania using the same technique (Kovacova *et al*. 1996). In addition, the seroprevalence of SFG rickettsiae reported herein in cattle (64.4%) is higher than that reported for cattle (9.6%) in Japan and for cattle (30%) from the north Zimbabwe, but lower than that reported in cattle from southern Zimbabwe in which almost 100% of 52 cattle tested were found to have antibodies reactive with *R. conorii* (Jilintai *et al*. 2008) (Kelly *et al*. 1991). Furthermore, our results were higher than those reported in Kenya in which the seroprevalence was found to be 43% in goats, 23% in sheep and 1% in cattle (Maina *et al*. 2014). These differences in seroprevalence may reflect geographic, ecologic and climate differences that affect the diversity, abundance and distribution of ticks found in a particular area or region.

In this study, gender as expected, seemed to be irrelevant to the prevalence of SFG rickettsia antibodies in both cattle and goats. On the contrary, it was significantly higher (*P* < 0.05) in female compared with male in sheep. This may reflect a higher degree of attractiveness of females to tick vectors (Elhassan *et al*. 2014), but could be due to the continued replacement of males in the farms compared with females so that females were probably exposed to several tick seasons (Palmer *et al*. 1998). In cattle, unlike sheep and goats, seasons seemed to be relevant to seroprevalence of SFG rickettsiae antibodies. The prevalence was significantly (*P* < 0.05) higher in winter season. This could be attributed to the crowding of cattle together for warmth which will increase the possibility of contact to ticks and infection transmission. In this study, breeds seemed to be irrelevant to the prevalence of SFG rickettsia antibodies in cattle, sheep and goats (data not shown). With respect to geographic location only, the prevalence in goats was significantly (*P* < 0.05) higher in Khartoum North compared to the other two locations. The reasons of this situation could be due to the small sample size, but most of the goat samples from Khartoum North location were collected from intensively managed farms with poor tick control programmes. The prevalence of seropositivity was significantly (*P* < 0.05) higher in older animals compared with young ones in both sheep and goats. This may be due to maternal immunity transfer protecting young animals. Protection will decrease with animals’ ages and thus adult animals will have more exposure to infection and a higher prevalence (Jegede *et al*. 2015). However, there was no significant difference (*P* > 0.05) between different age groups in cattle.

The results obtained indicated that high prevalence of SFG rickettsiae antibodies in Khartoum State may pose a serious health problem. The results to the best of our knowledge represent the first serological detection of SFG rickettsiae in animals in Khartoum State. The seroprevalence of SFG in the three animal species reported herein might have been underestimated due to small numbers tested for each animal species. Ideally more than 300 animals for each species have been tested. In addition, due to limitations of study design, the question of domestic ruminants acting as a reservoir could not be answered definitively. Therefore, further investigations are urgently needed to screen domestic ruminant's sera over a longer period for possible rickettsaemia. These investigations are also needed in other parts of the country, in order to obtain more reliable data with regard to ruminants’ role in maintaining rickettsial natural life cycles. On the other hand, further investigations including human and ticks to assess the actual risk of rickettsial diseases in the country is required to implement necessary therapeutics and control.

## Source of funding

This study was funded by Bank of Animal Resources, Khartoum, Sudan.

## Conflict of interest

The authors declare that they have no conflict of interest.

## Contributions

Nagwa M. Eisawi drafted the manuscript; Nagwa M. Eisawi and Mohammed O. Hussien carried out the IFA assays; Azza B. Musa done the statistical analysis; Abdel Rahim M. El Hussein and Dina A. Hassan contributed to the concept, design and supervision of the study as well as revision of the manuscript.
